# Naive independent directors, corporate governance and firm performance

**DOI:** 10.3389/fpsyg.2022.984661

**Published:** 2022-09-02

**Authors:** Gaocai Chen, Xiangyu Chen, Peng Wan

**Affiliations:** ^1^School of Business, Anhui University, Hefei, China; ^2^School of Accounting, Zhejiang University of Finance and Economics, Hangzhou, China; ^3^School of Accounting, Zhejiang Gongshang University, Hangzhou, China

**Keywords:** naive independent directors, corporate governance, firm performance, tunneling, China

## Abstract

This paper mainly explores the effect of naive independent directors on firm performance. Using hand-collected data on Chinese listed companies, this study finds that the proportion of naive independent directors is positively associated with firm performance, and an increased proportion of naive independent directors reduce the probability of tunneling of controlling shareholders and financial distress. The findings are robust after using alternative explanatory variables and retro-causality tests. Furthermore, the relation between naive independent directors and firm performance mainly existed in firms with lower shareholdings of the largest shareholder and firms with lower financial leverage. Moreover, this paper finds that firm size, corporate ownership type, and equity balance degree are important factors affecting the appointment of naive independent directors. This paper offers further empirical evidence to the existing research related to naive independent directors and provides an effective way to improve corporate board governance.

## Introduction

China Securities Regulatory Commission (CSRC) established the independent director system in China’s listed companies in 2001. In order to ensure the proper function of the independent director system in China, relevant regulations have been made on the professional background of independent directors, remuneration restrictions, the number of part-time directorships, meeting attendances, issuance of independent opinions, and tenure of office. However, in the course of the operation of the independent director system, there are still unhealthy phenomena like “official directors,” which are questioned and denounced by the public. To solve this problem, the Organization Department of the Communist Party of China issued a regulation on 19 October 2013 (hereafter No. 18 document), which explicitly stipulates that incumbent party and government officials should not hold part-time positions in enterprises, and strictly restricts the part-time positions of departing officials in enterprises, leading to a large-scale flash quit of “official directors” in A-share listed companies (Liu et al., 2018). Furthermore, a notice from the General Office of the Ministry of Education further stipulated that university leaders at or above the deputy-director level are not allowed to take positions as independent directors in listed companies. As a result, there was another flash quit wave of “academic independent directors” in the A-share market within a short period of time (Chen et al., 2019). Since then, a great number of naive independent directors (hereafter referred to as Naives) have entered the board of directors of firms to fill the vacancies caused by the leavings of “official directors” and “academic independent directors.”

After the resignation of “official directors” and the flash quit of “academic independent directors,” the proportion of Naives on corporate boards further increased. By 2018, the proportion of Naives to the number of independent directors has risen to 43% in China. According to a 2013 report by Spencer Stuart, an American headhunting company, Naives account for approximately one-third of the new directors on boards of directors in American firms, and United States companies are increasingly willing to recruit Naives to their boards. The rising ratio of Naives triggers the research interest in whether Naives exert a similar positive effect on improving corporate governance and firm performance.

Although naive independent directors are increasingly common on corporate boards, scholars have rarely studied the impact of Naives on companies. Few studies, such as Kang et al. (2016), take American companies as an example and find that Naives can improve the board functioning, thus increasing the firm value. However, the channels by which naive independent directors exert an influence on board governance and hence firm performance have not been clearly investigated. This study explores the monitoring role of Naives by examining whether an increased proportion of naive independent directors can reduce the probability of tunneling of controlling shareholders and financial distress.

Using manually collected data on Chinese listed companies from 2008 to 2018, this paper explores the influence of naive independent directors on firm performance. It is found that the proportion of naive independent directors is positively related to firm performance, and an increased proportion of naive independent directors reduces the probability of tunneling of controlling shareholders and financial distress. The findings are robust after using alternative explanatory variables and retro-causality tests. Furthermore, the relation between naive independent directors and firm performance mainly exists in firms with lower shareholdings of the largest shareholder and firms with lower financial leverage.

This paper contributes to the literature as follows. First, this paper contributes to the studies regarding the monitoring role of naive directorship. Although the monitoring role of outside directors has been examined by Beasley (1996); Nguyen and Nielsen (2010), Sharma (2011); Bulathsinhalage and Pathirawasam (2017), the monitoring role of naive directorship has not been clearly addressed. Naive independent directors, as a specific kind of independent directors, may play a similar monitoring role as the skilled independent directors. Kang et al. (2016) provide the initial evidence that naive independent directors can improve a firm’s performance by enhancing the board governance. However, whether an increased proportion of naive independent directors can reduce the probability of financial distress and hence improve firms’ performance has not been examined, although Stichhauerova et al. (2020) provide this cluster effect in some industries in the Czechia.

Second, this paper provides a novel approach to improving corporate governance and firm performance. This study finds that in order to gain a good professional reputation, naive independent directors tend to be more conscientious and perform their monitoring duties better, thus improving the governance level of their companies, which is conducive to corporate performance. Based on the advantages of naive directors, firms should employ naive independent directors as an effective way to improve corporate governance, this effect is also similar to novice internationalization in the post-transition economy of Poland (BarłoŻewski and Tra̧pczyński, 2021).

Third, this paper uses China’s unique institutional background to carry out empirical research, enriching relevant literature in the field of independent directors. Following the regulation issued by the CPC Central Committee in 2013 and the notice from the General Office of the Ministry of Education in 2015, the resignation of “official directors” and flash quit of “academic independent directors” led to a significant increase in naive independent directors, and the proportion of Naives to the number of independent directors has risen to 43% by 2018. The rapid rising ratios of Naives in China make it serve as an ideal setting to test the governance function of naive directors, and the monitoring role of Naives will be cleaner.

The structure of the remaining paper is as follows. The second section is the literature review, and the theoretical framework and hypothesis development consist the third section of this paper. Data and methodology is the fourth section, and the fifth section is empirical results and analysis. Cross-sectional analysis and further analysis form the sixth and seventh parts of this paper respectively, and the last section is conclusions and limitations.

## Literature review

Research on naive independent directors has focused on the governance characteristics of Naives. Scholars generally agree that naive independent directors tend to work hard and thus gain a good market reputation (e.g., Tran, 2015; Xue, 2019). According to Holmstrom’s (1982) career concern model, at the beginning of their careers, agents with an awareness of career development will work hard to build their reputations. Yermack (2004) finds that most outside directors who are new to the company build their reputation solely on their current or previous work. Kang et al. (2016) argue that Naives play an active role in monitoring regardless of their age. Therefore, independent board members who are new to the board market try to work as hard as possible to gain a good reputation, and in turn, a good reputation brings about more opportunities for Naives. Existing studies also find independent directors with an awareness of career development are more likely to challenge or oppose existing management and subsequently gain more board seats in the director market (Dewally and Peck, 2010; Jiang and Kim, 2015). Levit and Malenko (2016) show that independent directors in the early stages of their careers work harder as a way to gain more directorships, higher prestige and salary, and better social connections.

The frequency of board meetings, a reflection of the active degree of the board of directors, and independent directors’ active participation in board meetings, is beneficial for them to communicate with other board members, accumulate management experience, and provide suggestions and supervision for corporate management (Chou et al., 2013; Liang et al., 2013). Chen et al. (2022) argue that Naives with higher career concerns work more diligently and therefore are more willing to attend board meetings compared with seasoned independent directors. Other literature suggests that board attendance exerts a positive effect on firm performance. A study by Masulis and Mobbs (2014) finds that independent directors, taking positions in multiple listed companies, allot their time and energy based on the relative reputation of the board seats. When the reputation of the director’s seat is higher, the frequency of directors’ participation in board meetings increases, and firm performance improves accordingly. Independent directors’ active participation in board meetings also brings considerable reputation and financial rewards to them. In the practice of Chinese listed companies, according to Jiang and Kim (2015), independent directors who actively participate in board meetings and perform advisory and supervisory duties are rewarded with more positions in the future. Therefore, from an individual’s perspective, Naives are less inclined to be absent from board meetings, and hence improve the firm’s performance. Firms with more preoccupied independent directors who attend fewer meetings have declining firm valuation and operating performance and exhibit weaker merger and acquisition (M&A) profitability and accounting quality (Masulis and Zhang, 2019).

Some studies have examined the roles of independent directors in other countries. In Africa, Darko et al. (2016) focus on the effect of director and board structure on corporate performance in Ghana, Ngwakwe et al. (2014) investigate the independent directors and corporate sustainability in South Africa and Nigerian. In Asia, Nguyen et al. (2017) have probed into the relationship between independent directors and corporate performance in Viet Nam, Hossain and Oon (2022) compare the effect of independent directors on corporate performance in German and Indonesian listed firms, Nor and Rahman (2019) provide the supporting evidence from Malaysia. In European Union, Garcia-Ramos and Garcia-Olalla (2014) find empirical shreds of evidence from Southern Europe, Merendino and Melville (2019) find independent directors do have a non-linear effect on performance in Italy. Tran (2021) examines how independent directors affect corporate performance by using an international data set of 1,817 firm-year observations in 20 countries across Asia, America, and Europe.

## Theoretical framework and hypothesis development

### Naives and firm performance

Fama (1980); Fama and Jensen (1983) claim that the key function of a board of directors (especially outside directors) is to supervise and manage personnel to ensure that they act in the best interests of shareholders. Fama and Jensen (1983) hold that the board of directors has three main functions: monitoring, advising and approving plans, evaluating the performance of senior managers, and rewarding or punishing managers. Of these, inside directors (corporate executive officers) primarily provide valuable information about the company’s activities and play an advisory role, while outside directors can provide advice and oversight in evaluating the decisions of corporate executives (Chen et al., 2020). To conclude, an important function of independent directors is to provide supervision to the board of directors and the management. Thus, Naives may play a stronger supervisory role because they have not yet established ties with the board. Proposing a management-friendly model, Core et al. (1999) suggest that independent directors may become assimilated by management, and their supervisory role decreases with the extension of their term of office. According to the model, Naives are better supervisors than seasoned independent directors. Kang et al. (2016) emphasize that Naives facilitate the sensitivity to CEO remuneration and mobility and that they play an important monitoring role in setting CEO pay and incentives. However, Jiang et al. (2010) argue that the highly concentrated ownership of listed companies in China leads to the main agency conflicts occurring between minority shareholders and controlling shareholders, and therefore a major goal of corporate governance is to prevent the property of minority shareholders from being misappropriated. The main supervisory function of independent directors is to protect the interests of minority shareholders from being infringed by controlling shareholders.

According to the reputation hypothesis proposed by Fama and Jensen (1983), although independent directors receive less pay for their work, they are less likely to collude with management for their own reputation maintenance. Therefore, independent directors actively undertake their responsibilities. Specifically, the source of their motivation to work is reputation incentive, which lays a good foundation for the implementation of the independent director system. Jiang et al. (2016) argue that the reputation incentive of independent directors can be also reflected in their voting behavior. García-Sánchez and Martínez-Ferrero (2018); Khoo et al. (2022) find the effect of the reputation incentives on corporate social responsibility performance, while Yang et al. (2022) investigate the influence of this incentive on pay-for-performance in China, Le et al. (2022) investigate the association between independent directors’ reputation incentives and firm performance using Australia’s top 500 listed firms for 2004−2019. There are mainly two ways to help independent directors build a reputation: on the one hand, the professional skills that they possess, including research in a specific field or being an expert in a certain field. On the other hand, independent directors establish a reputation while performing their job. Reputation is a symbol of high social status for an independent director. Moreover, a good reputation can bring economic benefits and social resources to independent directors, so they tend to actively maintain their reputation (Bryan and Mason, 2020). If their reputation is damaged, the future interests of independents director are also ruined. The damage is often unrepairable. When a naive independent director first enters the director labor market, he or she is very concerned about the reputation acquired from his or her work as an independent director. At this time, they typically take their responsibilities seriously, actively participate in board meetings, and hold fewer concurrent positions to focus on their current job. Previous research also documents that directors’ concern about reputation affects corporate governance and thus corporate value (Lel and Miller, 2019). All in all, Naives makes efforts to provide suggestions and improve board governance and firm performance to gain a good reputation.

Therefore, this paper argues that the appointment of naive independent directors has an impact on the board governance structure, which in turn affects firm performance. Being inexperienced and incompetent in the director labor market, Naives have more incentives to actively participate in corporate affairs and enhance their oversight over the board compared with seasoned independent directors. The main objective of the introduction of the independent director system in China is to enhance the monitoring of management and major shareholders. The increase in the proportion of naive independent directors will significantly enhance their monitoring ability and hence improve firm performance. In summary, this paper proposes the hypothesis H1:

**H1:** The proportion of Naives is positively associated with firms’ financial performance, ceteris paribus.

### Naives and tunneling of controlling shareholders

Previous studies show that independent directors in Chinese listed companies mainly play a monitoring role. Therefore, this paper explores the channels for Naives to improve board governance from a monitoring perspective.

#### Naives and tunneling of controlling shareholders

With the development of the modern enterprise system, the core issue of corporate governance has gradually shifted from the principal-agent conflict between shareholders and managers to the conflict of interests between major shareholders and small and medium shareholders. The main purpose of CSRC’s introduction of the independent director system in 2001 aims to strengthen the independence of the board of directors in listed companies, protect the legitimate rights and interests of small and medium shareholders, and solve the agency problem arising from one share dominance. Jiang et al. (2010) reveal that in China, corporate resources are often diverted by the controlling shareholders from listed forms to their other entities (most of which are unlisted) employing inter-corporate loans which are generally reported on the balance sheets of lending firms under the accounting item “Other receivables.” Since 2006, the CSRC has demanded companies disclose the actual number of controlling shareholders’ inter-corporate loans. However, Qian and Yeung (2015) point out that the number of companies with non-zero other receivables has been increasing, indicating that the tunneling practice remains widespread although it seems that CSRC’s regulation has reduced the magnitude of tunneling of large shareholders. Therefore, this paper proposes hypothesis H2:

**H2:** The proportion of Naives is significantly and negatively associated with other receivables, ceteris paribus.

### Naives and financial distress

Public companies may be at high risk due to lack of resources and uncertainty of internal and external environment in the early stage, and fierce competition in the late stage. Durana et al. (2021) clarify the impact of corporate life cycle and bankruptcy on earnings management based on the sample of Central European companies from 2015 to 2019, in order to describe the behavior of companies at different stages of the corporate life cycle. Therefore, risk avoidance is extremely critical to the survival and growth of listed companies. But for listed companies, risks are everywhere, including operational risks and financial risks, etc. Too many risks may increase bankruptcy probability, Krulicky and Horak (2021) propose artificial neural networks should determine the ability of a corporate to survive potential financial distress in the Czechia. An increase in the bankruptcy probability reflects risks existing in a company. Jiang et al. (2010) reveal that firms with high ORECTA (%) (other receivables scaled by total assets) are more prone to experience poor operating performance and confront financial distress in the future, Liu et al. (2021) use this variable proxying for the degree to which a supplier implements supply chain finance (SCF), examining the relationship between SCF, performance, and risk. Hence, an increase of naive independent directors, who perform a supervisory function and monitor management’s business behavior and decisions, may lower the probability of financial distress and the risk of bankruptcy. Therefore, this paper proposes hypothesis H3:

**H3:** The proportion of Naives is significantly and negatively related to financial distress, ceteris paribus.

## Data and methodology

### Data

The research sample of this paper includes A-share listed companies (2008−2018) in CSMAR, CNRDS, and Sinofin databases. The sample of naive independent directors at the firm level is 20732 observations, including 2729 companies. The sample is initially screened in terms of the following criteria: (1) companies in finance industries are excluded; (2) ST firms or *ST firms are excluded; (3) companies undergoing major restructuring in the current year are excluded, for the restructuring may lead to significant changes in several financial indicators of the companies; (4) lacking data to calculate main variables.

### Variable measurement

#### Firm performance

Firm performance is the starting point and core of corporate governance. The measures of firm performance can be generally divided into two categories: market performance and financial performance. The current financial performance evaluation is based on the DuPont System of Financial Analysis. Financial performance, including the solvency, profitability, cash flow, and the ability of assets operation, is often an evaluation of the short-term financial performance of enterprises and a reflection of the firm performance in the past. ROE (return on equity), with strong comprehensive strength, is used to reflect the capital earning capacity of a firm. However, it may not truly reflect the actual profitability of a firm due to entrusting financing and asset restructuring of many listed companies. Based on existing empirical studies, Tobin Q is adopted to measure the market performance of firms, and ROA (return on assets) is adopted to measure the financial performance of firms. Drawing on the relevant study of Liu Y. et al. (2015) in the robustness test, this study uses ROS (return on sales) and EBIT (earnings before interest and taxes/total assets) as proxies to measure firm performance.

#### Tunneling of controlling shareholders

According to Jiang et al. (2010), corporate resources are often diverted from listed firms to controlling shareholders’ other entities through inter-corporate loans reporting under “Other receivables” on the balance sheet of the lending firms. However, other scholars are more inclined to use earnings management in a transition economy, Valaskova et al. (2021a) provide supportive evidence from Central European. Therefore, this paper chooses other receivables (%) to measure the encroachment of interests of controlling shareholders on small and medium shareholders. Other receivables (%) are equal to other receivables divided by total assets.

#### Financial distress

Indicators in previous studies to measure bankruptcy risk include whether a company is an ST company and its changes in firm performance etc. The most commonly used indicator is the Altman-Z index. In this study, ST companies are excluded, and Altman-Z is used to measure the financial distress of companies, thus measuring the monitoring function of independent directors.

#### Explanatory variables

Following Kang et al. (2016); Chen et al. (2022), this paper classifies independent directors into naive independent directors and seasoned independent directors according to their board experience. Naives, independent directors with less than three years of board experience or experienced one term of office are set as 1, while seasoned independent directors, the ones with three or more years of board experience, are set as 0.

This paper uses executive tenure information and collates it by hand in the following ways: (1) companies in banking, finance, insurance industries, ST, *ST, etc., are excluded; (2) based on the list of companies, the period of executive tenure data is selected from 1999 to 2018 since the earliest period of executive information provided by CSMAR is 1999; (3) according to the executive tenure data, a total of 14,707 independent directors who served in the list of companies after 2008 are screened out, and based on this list, the independent directors are manually collated from 1999 to 2018 during their tenure as executives in listed companies. The manual collation method can check the errors of information disclosed by listed companies and can distinguish different independent directors with the same name based on other information; (4) after collating the tenure of independent directors, the tenure of independent directors before 2008 is calculated and dummy variables are set, if the tenure is less than 3 years before 2008, then in 2008, the independent director is naive. The years 2009−2018 are calculated by this method backward, and finally, the naive data for the period 2008−2018 are obtained.

### Regression model

Following the previous studies, this paper constructs the following model to test the hypotheses H1−H3.


(1) Y=β+0βNaive1+Controlvariables+Industry+Year+e

Where Y represents Tobin Q, ROA, Occupy, and Altman-Z depends on which hypothesis will be examined respectively. The paper also controls the regular variables such as firm size (Size), board size (Bnum), the proportion of female independent directors (Female), firm’s age (Firma), equity concentration (First), whether the Chairman and CEO are the same people (Dual), the proportion of independent directors (Inde), sales growth rate (Growth), Chairman’s age (Age), Chairman’s gender (Gend), and Chairman’s education degree (Degree). Table 1 shows the definitions and algorithms of these variables. This paper also controls for the industry and firm fixed effect.

**TABLE 1:** Variable definition.

Variable name	Variable symbol	Variable calculation
Tobin Q	Tobin Q	Market value/total assets
Return on assets	ROA	Net profit/total assets
Other receivables	Occupy	Other receivables/total assets
Financial distress	Altman-Z	0.012*Total working capital assets*100/Total assets + 0.014*Retained earnings*100/Total assets + 0.33*Earnings before interest and taxes*100/Total assets + 0.006*Total market value of stocks*100/Book value of liabilities + 0.0099*Sales income *100/total assets
Naive director (%)	Naive	The proportion of Naive independent directors in the total number of independent directors
Firm size	Size	The natural logarithm of the company’s total assets
Board size	Bnum	Natural logarithm of the number of board members
The proportion of female independent directors	Female	The proportion of female directors in the number of independent directors
Firm age	Firma	The natural logarithm of listed years of a company
Equity concentration	First	The largest shareholder’s shareholding ratio
Dual positions	Dual	If the chairman is also the general manager, the dummy variable is equal to 1, otherwise, it is 0.
The proportion of independent directors	Inde	The ratio of independent directors to the number of directors
Growth	Growth	The average sales growth rate in the past three years in year t
Age	Age	Chairman’s age
Gender	Gend	Chairman’s gender
Education background	Degree	Chairman’s education degree

## Empirical results and analysis

### Descriptive statistics

There are 20,732 observations in total. As shown in Table 2, Tobin Q and ROA are included in the firm performance indicators. The minimum value of Tobin Q is 0.88, the maximum value is 10.73, and the standard deviation is 1.7. The difference is significant. The minimum value of ROA is −0.16, the maximum value is 0.22, and the standard deviation is 0.06. Monitoring proxy variables include Occupy and Altman-Z. Occupy has a minimum value of 0 and a maximum value of 0.42, with a standard deviation of 0.03. The difference is small. Altman-Z has a minimum value of 0.91, a maximum value of 40.74, a mean value of 5.82, a median value of 3.83, and a standard deviation of 6.19. The sampling variation is large, indicating an uneven data distribution.

**TABLE 2:** Descriptive statistics.

Variables	Obs.	Mean	Median	*SD*	Min	Max
Tobin Q	20265	2.49	1.95	1.70	0.88	10.73
ROA	20731	0.05	0.04	0.06	–0.16	0.22
Occupy	20415	0.07	0.03	0.09	0.00	0.42
Altman-Z	19001	5.87	3.87	6.21	0.91	40.74
Naive	20732	0.48	0.50	0.34	0.00	1.00
First	20729	0.36	0.34	0.15	0.09	0.75
Size	20732	22.07	21.90	1.25	19.63	26.02
Bnum	20732	2.15	2.20	0.20	1.61	2.71
Female	20732	0.16	0.00	0.20	0.00	0.67
Firma	20732	2.87	2.89	0.30	1.97	3.54
Dual	20732	0.27	0.00	0.44	0.00	1.00
Inde	20732	0.37	0.33	0.05	0.33	0.57
Growth	20732	0.23	0.13	0.49	–0.24	3.88
Age	20732	5.28	5.30	0.70	3.60	7.20
Gend	20732	0.05	0.00	0.21	0.00	1.00
Degree	20732	2.73	3.00	1.61	0.00	5.00

As is shown in Table 3, the average presence of naive directors decreased year by year from 2009 to 2013, and then increased from 2014 to 2017. From 2013 to 2014, the average presence of naive directors increased by 4%. This may be due to the release of the No. 18 document in 2013, which imposed a series of strict restrictions on the party and political leaders and cadres taking part-time jobs in firms, triggering a wave of resignations by politically connected independent directors. Additionally, the average presence of naive directors increased by 5% from 2015 to 2016. This may be due to the fact that in late 2015, the Ministry of Education issued a document requesting that leading cadres of universities at or above the deputy department level should not be appointed as independent directors of listed companies, which led to a flash quit of a large number of academic independent directors. Due to the departure of politically connected independent directors as well as academic independent directors, a large number of naive directors entered listed companies. Overall, a minimum value of the naive directors is 0, a maximum value of 1, an average value of 0.48, a median of 0.5, and a standard deviation of 0.34. The sample data is stable and conforms to normal distribution. The average presence is 48%, indicating that the number of naive directors equals that of seasoned independent directors, proving the importance of this study.

**TABLE 3:** Yearly descriptive statistics of explanatory variables.

Year	Obs.	Mean	Median	*SD*	Min	Max
2008	1071	0.5	0.5	0.32	0	1
2009	1120	0.55	0.67	0.31	0	1
2010	1262	0.54	0.6	0.31	0	1
2011	1604	0.48	0.4	0.34	0	1
2012	1865	0.41	0.33	0.34	0	1
2013	1982	0.39	0.33	0.34	0	1
2014	2088	0.43	0.33	0.34	0	1
2015	1936	0.49	0.5	0.33	0	1
2016	2230	0.53	0.67	0.33	0	1
2017	2573	0.54	0.67	0.33	0	1
2018	3001	0.47	0.33	0.34	0	1
Total	20732	0.48	0.5	0.34	0	1

### Correlation analysis

From the correlation coefficient matrix in Table 4, it can be seen that the correlation coefficient between the presence of naive directors and Tobin’s Q and ROA is significantly positive, initially indicating that an increase in naive directors is conducive to improving firm performance. The correlation coefficient between the presence of naive directors and the wealth expropriation of controlling shareholders (Occupy) is significantly negative, which tentatively indicates that naive directors can inhibit tunneling by controlling shareholders. The correlation coefficient between naive directors and the Altman-Z index is positive, and the higher the Altman-Z value, the lower the risk of corporate bankruptcy, indicating that an increase in naive independent directors can strengthen the monitoring role of independent directors and thus improve firm performance, which is consistent with the hypothesis of this study.

**TABLE 4:** Pearson correlation coefficient.

	TobinQ	ROA	Occupy	AltmanZ	Naive	First	Size	Bnum	Female	Firma	Dual	Inde	Growth	Age	Gend	Degree
**TobinQ**	1															
**ROA**	0.295***	1														
**Occupy**	−0.093***	−0.164***	1													
**AltmanZ**	0.666***	0.311***	−0.130***	1												
**Naive**	0.095***	0.018**	−0.028***	0.052***	1											
**First**	−0.065***	0.098***	−0.084***	−0.077***	−0.024***	1										
**Size**	−0.464***	−0.024***	0.115***	−0.398***	−0.109***	0.206***	1									
**Bnum**	−0.175***	0.018***	−0.036***	−0.148***	−0.042***	0.026***	0.256***	1								
**Female**	0.017**	0.006	0.008	0.012*	0.052***	−0.039***	−0.050***	−0.049***	1							
**Firma**	−0.071***	−0.083***	0.168***	−0.061***	−0.053***	−0.158***	0.183***	0.003	0.056***	1						
**Dual**	0.101***	0.038***	–0.002	0.094***	0.044***	−0.058***	−0.131***	−0.183***	0.026***	−0.026***	1					
**Inde**	0.063***	−0.027***	0.034***	0.032***	0.006	0.040***	0.013*	−0.506***	−0.014**	−0.030***	0.121***	1				
**Growth**	0.016**	0.100***	–0.007	−0.065***	0.011	0.038***	0.106***	−0.017**	–0.009	0.033***	–0.007	0.023***	1			
**Age**	−0.048***	0.042***	−0.025***	0.007	−0.040***	0.01	0.113***	0.069***	0.022***	0.140***	−0.110***	−0.050***	−0.058***	1		
**Gend**	0.019***	0.023***	0.024***	0.020***	0.008	0.004	−0.019***	−0.038***	0.009	0.022***	0.020***	0.026***	–0.004	−0.037***	1	
**Degree**	0.038***	0.045***	−0.030***	0.044***	−0.016**	−0.053***	0.014**	–0.004	−0.013*	−0.077***	0.097***	0.007	−0.050***	−0.069***	–0.004	1

*, **, *** denote significance level for two-tail test at 10%, 5%, and 1%, respectively.

### Multiple regression analysis

#### Naive directors and firm performance

Table 5 exhibits the regression results of naive independent directors, Tobin Q and ROA. The regression coefficient between the proportion of naive independent directors and the market performance indicator Tobin Q is 0.091 and the *t*-value is 3.46, which is significant at the confidence level of 1%. The regression coefficient between the proportion of naive independent directors and ROA is 0.002 and the *t*-value is 1.73, which is significant at the confidence level of 10%. It suggests that the participation of naive independent directors does promote corporate performance, and hypothesis H1 is supported. The results suggest that being inexperienced and incompetent in the director labor market, Naives have more incentives to actively participate in corporate affairs and enhance their oversight over the board compared with seasoned independent directors. The increase in the proportion of naive independent directors indeed significantly enhances their monitoring ability and hence improves firm performance.

**TABLE 5:** Regression results of naive directors and firm performance.

	Tobin Q	ROA
*naïve*	0.091*** (3.46)	0.002* (1.73)
*Size*	−0.503*** (−63.80)	−0.002*** (−6.52)
*Bnum*	0.033 (0.60)	0.006*** (2.75)
*Female*	−0.144*** (−3.17)	0.003 (1.50)
*Firma*	0.214*** (6.93)	−0.0123*** (−9.4)
*First*	0.988*** (16.03)	0.036*** (13.80)
*Dual*	0.106*** (5.18)	0.006*** (6.40)
*Inde*	1.257*** (6.35)	−0.027*** (−3.21)
*Growth*	0.204*** (11.19)	0.013*** (16.18)
*Age*	0.006 (0.45)	0.006*** (9.98)
*Gend*	0.039 (0.95)	0.007*** (3.99)
*Degree*	0.006 (1.05)	0.002*** (7.64)
Intercept	9.465*** (43.33)	0.073*** (7.77)
Industry Year	Control Control	Control Control
N	20263	20728
Adj-R^2^	0.194	0.036
*F*-value	408.226***	65.79***

*, *** denote significance level for two-tail test at 10%, 5% and 1%, respectively.

#### Naive directors and other receivables

As is shown in Column (1) of Table 6, the regression coefficient between the proportion of naive directors and other receivables (Occupy) is −0.004 and the *t*-value is −2.09, indicating that the presence of naive directors is negatively associated with the tunneling activities by controlling shareholders. In other words, naive directors are capable of inhibiting the tunneling by controlling shareholders. The result is consistent with hypothesis H2. The result indicates that naive directors have a monitoring role when constraining the tunneling practice of large shareholders.

**TABLE 6:** Regression results of Naives and the tunneling and financial distress.

	Occupy (1)	Altman-Z (2)
*naïve*	−0.004**	0.319**
	(−2.09)	(2.54)
*Size*	0.010***	−1.923***
	(17.52)	(−51.06)
*Bnum*	−0.025***	−0.951***
	(−6.77)	(−3.69)
*Female*	0.001	−0.333
	(0.18)	(−1.58)
*Firma*	0.039***	0.347**
	(18.84)	(2.40)
*First*	−0.051***	0.712**
	(−12.08)	(2.46)
*Dual*	−0.001	0.490***
	(−0.82)	(4.98)
*Inde*	0.011	2.370**
	(0.82)	(2.56)
*Growth*	−0.005***	−0.317***
	(−4.14)	(−3.76)
*State*	−0.003**	−0.438***
	(−2.53)	(−4.78)
*Age*	−0.007***	0.561***
	(−8.36)	(9.28)
*Gend*	0.007***	0.289
	(2.64)	(1.49)
*Degree*	−0.002***	0.192***
	(−4.59)	(7.44)
Intercept	−0.141***	44.875***
	(−9.18)	(42.79)
Industry	Control	Control
Year	Control	Control
N	20412	18999
Adj-R^2^	0.051	0.171
*F*-value	85.123***	301.539***

**, *** denote significance level for two-tail test at 5%, and 1%, respectively.

#### Naive directors and financial distress

As is shown in Column (2) of Table 6, the regression coefficient between the proportion of naive directors and the Altman-Z value is 0.319, with a *t*-value of 2.54, which is statistically significant at the 5% confidence level. Since the Altman-Z value is a negative indicator, that is, the lower the Altman-Z value of a firm, the lower the bankruptcy risk, indicating that the presence of naive directors reduces the possibility of the company falling into financial difficulties and then going bankrupt. Therefore, the result is consistent with hypothesis H3. The result indicates that naive directors play a supervisory function and monitor management’s business behavior and decisions, which lower the probability of financial distress and the risk of bankruptcy.

### Robustness tests

#### Alternative measures of firm performance

Table 7 demonstrates the regression results of Naives and the alternative measures of firm performance. ROS and EBIT, standing for return on sales and earnings before interest and tax respectively, are used as alternative measures for firm performance. The regression coefficients between the proportion of naive directors and ROS and EBIT are 0.006 and 0.005 respectively, with *t*-values of 3.33 and 2.65, both statically significant at the 1% confidence level. These robustness test results further validate the main findings of this study.

**TABLE 7:** Alternative measures of firm performance.

	ROS	EBIT
*naïve*	0.006***	0.005***
	(3.33)	(2.65)
*Size*	0.002*	0.013
	(1.69)	(8.37***)
*Bnum*	0.019***	0.021***
	(3.27)	(3.15)
*Female*	0.002	0.003
	(0.47)	(0.60)
*Firma*	−0.097***	−0.073***
	(−8.47)	(−5.42)
*First*	0.032***	0.043***
	(3.61)	(4.21)
*Dual*	0.004**	0.003
	(2.00)	(1.31)
*Inde*	0.000	0.021
	(0.02)	(1.05)
*Growth*	0.003**	0.001
	(2.41)	(0.53)
Intercept	0.277***	−0.014
	(5.99)	(−0.27)
Year	Control	Control
Industry	Control	Control
N	18515	18515
Adj-R^2^	0.624	0.665
*F*-value	8.144***	17.238***

*, **, *** denote significance level for two-tail test at 10%, 5%, and 1%, respectively.

#### Reverse causality

This study may suffer from endogeneity problems, as reverse causality may exist between the presence of naive independent directors and firm performance. For example, maintaining performance may bring changes in the effectiveness of board governance which hence leads to the appointment of naive directors. This paper re-estimates whether there is a reverse causality between the presence of naive directors and firm performance and monitoring functions. Table 8 exhibits the regression results of the indicators of firm performance and board governance in year t−1 and the presence of naive directors in year t. As can be seen from Table 8, Tobin Q, ROA, Occupy and Altman-Z are not significantly correlated with the presence of naive directors in year t−1. Therefore, there is no obvious evidence to support the reverse causality relationship in this paper, which alleviates the concern of endogeneity.

**TABLE 8:** Regression analysis of reverse causality.

	Naive			
*Tobin Q*	0.005			
	(1.56)			
*ROA*		−0.102		
		(−1.55)		
*Occupy*			0.001	
			(0.02)	
*Altman-Z*				−0.0002
				(−0.18)
Size	−0.017***	−0.015***	−0.017***	−0.017***
	(−5.17)	(−3.99)	(−5.08)	(−4.97)
Bnum	−0.005	−0.006	−0.005	−0.005
	(−0.26)	(−0.30)	(−0.23)	(−0.26)
Female	0.075***	0.076***	0.076***	0.075***
	(4.48)	(4.49)	(4.51)	(4.47)
Firma	−0.029**	−0.029**	−0.029**	−0.029**
	(−2.19)	(−2.18)	(−2.20)	(−2.19)
First	0.011	0.008	0.014	0.011
	(0.46)	(0.35)	(0.59)	(0.47)
Dual	0.008	0.008	0.009	0.008
	(1.06)	(0.99)	(1.08)	(1.06)
Inde	0.048	0.041	0.045	0.048
	(0.67)	(0.57)	(0.63)	(0.67)
Growth	0.016**	0.016**	0.017***	0.016**
	(2.51)	(2.42)	(2.65)	(2.52)
Age	−0.007	−0.007	−0.006	−0.007
	(−1.36)	(−1.38)	(−1.29)	(−1.35)
Gender	−0.008	−0.008	−0.007	−0.008
	(−0.48)	(−0.53)	(−0.42)	(−0.47)
Degree	−0.005***	−0.006***	−0.005***	−0.005***
	(−2.73)	(−2.76)	(−2.70)	(−2.72)
Intercept	0.946***	0.886***	0.940***	0.951***
	(10.23)	(8.83)	(10.16)	(9.89)
Year	Control	Control	Control	Control
Industry	Control	Control	Control	Control
N	9827	9827	9827	9827
Adj-R^2^	0.04	0.041	0.041	0.04
*F*-value	6.955***	7.144***	7.141***	6.958***

**, *** denote significance level for two-tail test at 5%, and 1%, respectively.

## Cross-sectional analysis

### The effects of the largest shareholder’s shareholding

The polarization of the shareholding ratio leads to the conflict between major shareholders and small and medium shareholders and the opposition to their interests. In the absence of a power balance with shareholder structure, major shareholders tend to sacrifice the interests of minority shareholders through infringement in order to maximize their own interests. The more concentrated the ownership of a company is, the more control the largest shareholder has over the company, the less constrained it is by other shareholders, and the easier it is to seek private interests by infringing the interests of other shareholders. Jiang et al. (2010) argue that controlling shareholders can effectively control the company even if their shareholding ratio is low. Therefore, when the controlling shareholders’ ownership is relatively low, the company still faces serious tunneling of the controlling shareholder. In short, the lower the shareholding of controlling shareholders, the less control they have, the more restrained when tunneling, and the less tunneling the company experiences. Therefore, Naives can monitor the interest encroachment of controlling shareholders to reduce the tunneling and thus improve the firm’s performance. Based on the above analysis, this paper expects that the relationship between naive independent directors and firm performance mainly existed in firms with lower shareholdings of the largest shareholder.

As shown in Table 9, the regression coefficient between naive directors and Tobin Q in the higher ownership group is −0.045 (*t* = −1.31) and the coefficient with ROA is −0.045 (*t* = −0.18), both being statistically insignificant. This indicates that an increase in naive directors does not improve firm performance when the largest shareholder’s ownership is high. Whereas, in the low ownership group, the regression coefficients between naive directors and Tobin Q and ROA are 0.112 and 0.003, with *t*-values of 2.67 and 1.66 which are significant at the 1% and 10% levels respectively. The results are consistent with the expectation that the proportion of naive directors is positively associated with firm performance when the shareholding of the largest shareholder is lower.

**TABLE 9:** The effects of the largest shareholders’ shareholdings.

	Tobin Q		ROA		Occupy		Altman-Z	
	Low shareholding	High shareholding	Low shareholding	High shareholding	Low shareholding	High shareholding	Low shareholding	High shareholding
*naïve*	0.112***	−0.045	0.003*	−0.001	−0.004*	0.002	0.027	0.176
	(2.67)	(−1.31)	(1.66)	(−0.18)	(−1.67)	(0.87)	(0.15)	(1.12)
*Size*	−0.776***	−0.498***	0.001	0.002***	0.011***	0.002***	−2.259***	−1.554***
	(−58.75)	(−43.24)	(1.15)	(4.66)	(14.48)	(3.05)	(−39.50)	(−29.29)
*Bnum*	−0.048	0.061	0.004	−0.001	−0.03***	−0.004	−0.70*	−0.685**
	(−0.52)	(0.86)	(1.17)	(−0.22)	(−5.27)	(−0.89)	(−1.83)	(−2.14)
*Female*	−0.176**	−0.120*	0.000	0.004*	0.004	0.001	−0.82***	−0.344
	(−2.55)	(−1.95)	(−0.16)	(1.74)	(0.90)	(0.29)	(−2.83)	(−1.26)
*Firma*	−0.16***	−0.054	−0.01***	−0.01***	0.04***	0.024***	0.302	−1.08***
	(−2.94)	(−1.26)	(−7.75)	(−7.84)	(12.62)	(9.41)	(1.25)	(−5.62)
*Dual*	0.169***	0.087***	0.002**	0.006***	0.003	−0.003	0.249*	0.682***
	(5.43)	(2.95)	(2.03)	(4.99)	(1.38)	(−1.33)	(1.86)	(5.12)
*Inde*	1.472***	1.063***	−0.028**	−0.026**	−0.035*	0.034**	1.081	0.411
	(4.57)	(4.20)	(−2.32)	(−2.45)	(−1.69)	(2.11)	(0.80)	(0.36)
*Growth*	0.201***	0.286***	0.011***	0.012***	−0.01***	−0.01***	−0.237*	−0.15
	(6.77)	(12.16)	(9.70)	(11.86)	(−4.42)	(−6.68)	(−1.90)	(−1.43)
*Age*	0.032	−0.007	0.005***	0.004***	−0.009***	−0.004***	0.476***	0.467***
	(1.57)	(−0.39)	(7.19)	(5.78)	(−6.71)	(−3.32)	(5.54)	(6.05)
*Gend*	0.04	0.076	0.004*	0.009***	0.008**	0.000	0.184	0.400
	(0.65)	(1.36)	(1.72)	(3.86)	(2.06)	(0.01)	(0.69)	(1.60)
*Degree*	0.013	0.030***	0.001*	0.001***	0.000	0.000	0.011	0.217***
	(1.50)	(4.14)	(1.76)	(4.16)	(−0.60)	(−0.16)	(0.29)	(6.68)
Intercept	19.66***	12.73***	0.002	−0.028**	−0.18***	−0.09***	54.40***	40.94***
	(50.64)	(41.08)	(0.11)	(−2.31)	(−7.40)	(−4.95)	(31.80)	(28.78)
Industry	Control	Control	Control	Control	Control	Control	Control	Control
Year	Control	Control	Control	Control	Control	Control	Control	Control
N	10040	10218	10363	10365	10230	10182	9197	9801
Adj-R^2^	0.483	0.391	0.019	0.027	0.042	0.014	0.295	0.263
*F*-value	371.048***	205.211***	18.99***	27.519***	38.196***	12.819***	157.87***	111.2***

*, **, *** denote significance level for two-tail test at 10%, 5%, and 1%, respectively.

The regressions of naive directors and tunneling by controlling shareholders and financial distress are conducted according to the ownership of the largest shareholder. In the high ownership group, the regression coefficients between naive directors and tunneling by controlling shareholders and financial distress are 0.002 and 0.176 respectively, and the *t*-values are 0.87 and 1.12 respectively, and the results are not significant, indicating that when the largest shareholder ownership is high, an increase in naive directors cannot reduce tunneling by controlling shareholders and financial distress. However, in the low ownership group, the regression coefficient between naive directors and tunneling by controlling shareholders is −0.004 with a *t*-value of −1.67, being statistically significant at the 10% confidence level. The regression coefficient between naive directors and financial distress is 0.027, with a *t*-value of 0.15. This indicates that the presence of naive directors is not related to financial distress. Taken together, when the largest shareholder’s ownership is low, an increase in naive directors can reduce tunneling by controlling shareholders and the result is consistent with the paper’s expectations.

### The effects of financial leverage

Previous studies (Kor and Sundaramurthy, 2008; Kim et al., 2014; Kang et al., 2016) suggest that naive independent directors usually lack social connections with other board members and they can access information through the contact of the board of directors. But this may have an impact on the effectiveness of their advisory and supervisory powers, reducing the value of the Naives to the company. Coles et al. (2008) argue that the more complex a company, the higher the request for independent directors’ expertise and information resources. Kang et al. (2016) also find that Naives play a less significant role in the corporate value of complex companies than non-complex companies. The complexity of a firm is distinguished by its financial leverage. If the financial leverage of a firm is higher than the industry average, it is classified as a complex firm; otherwise, it is a non-complex firm. This paper proposes that the proportion of Naives is significantly and positively related to firm performance and is significantly and negatively related to tunneling of controlling shareholders and financial distress only when firms with lower financial leverage.

This paper calculates the financial leverage as the total liabilities divided by the total assets and divides the full sample into two subsamples according to the median value of financial leverage for each industry and each year. The first four columns of Table 10 present group regressions of naive directors and the firm performance indicators (Tobin Q and ROA), respectively. The results show that in the high leverage group, the regression coefficient between naive directors and Tobin Q is −0.02, and the coefficient with ROA is 0.001, with *t*-values of −0.48 and −0.56, respectively, which are not significant. This indicates that in the higher leverage group, an increase in naive directors does not improve firm performance. In the lower leverage group, the regression coefficients between naive directors and Tobin Q and ROA are 0.140 and 0.003, with *t*-values of 3.90 and 1.79, respectively, which are statistically significant at 1% and 10% levels. The results are consistent with the expectation that the presence of naive directors is associated with firm performance positively when corporate financial leverage is lower., The last four columns of Table 10 exhibit the regression results of naive directors and the tunneling by controlling shareholders and financial distress according to the level of corporate leverage. Similarly, in the higher leverage group, the regression coefficients of naive directors and the tunneling by controlling shareholders and financial distress are not significant, indicating that an increase in naive directors cannot reduce the tunneling by controlling shareholders and financial distress when corporate leverage is high. On the contrary, in the lower leverage group, the regression coefficient between naive directors and the tunneling by controlling shareholders is −0.006 with a *t*-value of −2.48, being statistically significant at the 1% level. The regression coefficient between naive directors and Altman-Z is 0.509, with a *t*-value of 3.05, being statistically significant at the 1% level. This indicates that the presence of naive directors is negatively associated with the possibility of financial distress. By and large, the result is consistent with the expectation that an increase in naive directors can restrain the tunneling behavior of controlling shareholders and reduce the probability of the company falling into financial distress when financial leverage is relatively low.

**TABLE 10:** The effects of financial leverage.

	Tobin Q		ROA		Occupy		Altman-Z	
	Low leverage	High leverage	Low leverage	High leverage	Low leverage	High leverage	Low leverage	High leverage
*naïve*	0.140***	−0.020	0.003*	0.001	−0.006**	−0.001	0.509***	0.181
	(3.90)	(−0.48)	(1.79)	(0.56)	(−2.48)	(−0.24)	(3.05)	(0.97)
*Size*	−0.61***	−0.69***	−0.003***	−0.002***	0.012***	0.008***	−1.90***	−1.92***
	(−54.04)	(−50.62)	(−5.70)	(−3.19)	(14.91)	(9.83)	(−38.48)	(−33.33)
*Bnum*	0.112	0.01	0.004	0.009***	−0.032***	−0.021***	−0.662*	−1.286***
	(1.45)	(0.11)	(1.24)	(2.69)	(−5.76)	(−4.12)	(−1.88)	(−3.42)
*Female*	−0.205***	−0.09	0.002	0.003	−0.001	0.003	−0.615**	−0.046
	(−3.47)	(−1.24)	(0.79)	(1.25)	(−0.30)	(0.77)	(−2.26)	(−0.14)
*Firma*	−0.066	−0.081	−0.010***	−0.015***	0.037***	0.044***	0.12	0.588***
	(−1.41)	(−1.54)	(−5.11)	(−8.30)	(11.70)	(15.26)	(0.59)	(2.73)
*First*	0.720***	0.566***	0.046***	0.026***	−0.048***	−0.052***	1.021***	0.345
	(8.41)	(5.83)	(12.20)	(7.04)	(−7.79)	(−9.09)	(2.64)	(0.81)
*Dual*	0.111***	0.144***	0.002*	0.010***	0.003*	−0.006***	0.216*	0.902***
	(4.17)	(4.16)	(1.85)	(7.43)	(1.65)	(−2.89)	(1.76)	(5.70)
*Inde*	1.377***	1.493***	−0.023*	−0.034***	−0.037*	0.059***	1.305	3.533***
	(5.09)	(4.94)	(−1.85)	(−2.90)	(−1.85)	(3.23)	(1.05)	(2.59)
*Growth*	0.182***	0.330***	0.011***	0.015***	−0.006***	−0.005***	−0.157	−0.425***
	(7.33)	(11.66)	(9.83)	(13.47)	(−3.13)	(−2.96)	(−1.42)	(−3.37)
*Age*	0.035**	0.017	0.006***	0.005***	−0.009***	−0.005***	0.452***	0.700***
	(2.02)	(0.86)	(7.42)	(6.43)	(−7.23)	(−4.04)	(5.69)	(7.70)
*Gend*	0.049	0.097	0.005**	0.009***	0.010**	0.005	0.245	0.367
	(0.91)	(1.47)	(2.18)	(3.50)	(2.56)	(1.24)	(0.98)	(1.23)
*Degree*	0.019**	0.037***	0.001***	0.002***	−0.002***	−0.002***	0.082**	0.301***
	(2.43)	(4.28)	(4.26)	(6.52)	(−3.02)	(−3.12)	(2.38)	(7.85)
Intercept	14.86***	17.09***	0.078***	0.069***	−0.142***	−0.150***	45.40***	43.85***
	(45.20)	(46.14)	(5.66)	(5.23)	(−6.14)	(−7.17)	(31.34)	(28.22)
Industry	Control	Control	Control	Control	Control	Control	Control	Control
Year	Control	Control	Control	Control	Control	Control	Control	Control
N	10169	10090	10365	10363	10210	10202	9324	9675
Adj-R^2^	0.421	0.465	0.033	0.044	0.051	0.053	0.178	0.169
*F*-value	287***	258.591***	30.151***	40.311***	43.033***	44.844***	156.449***	152.214***

*, **, *** denote significance level for two-tail test at 10%, 5%, and 1%, respectively.

## Further analysis

In further analysis, this paper investigates the factors that affect the appointment of naive directors and which companies can benefit more from naive directors.

### Firm size and naive directors

From the perspective of firm size, the larger and more complex the firm, the more information and experience managers need in firm management and decision-making, including debt management (Valaskova et al., 2021b). Coles et al. (2008) claim that firms with complicated operation activities have a higher demand for knowledge, expertise, and information resources from independent directors. Kang et al. (2016) argue that naive directors tend to lack enterprise-wide experience and board network connections, which will further limit naive directors’ ability to access information. In conclusion, on the one hand, naive directors lack knowledge about listed companies; on the other hand, they do not have the relevant managerial experience to meet the needs of large listed companies. Therefore, the larger the listed company, the less likely it is to appoint naive directors.

In Table 11, the regression coefficient between firm size and naive directors is −0.026 with a *t*-value of −7.96, which is statistically significant at the 1% level, proving that firm size is negatively related to naive directors. This indicates that the larger and more complex a company is, the more it needs independent directors to provide consulting services, which is consistent with existing research. This is probably because the more complex the firm, the more internal and external information is required for business management and decision-making. It takes a long time for naive directors to understand the business model of firms and to accumulate extensive experience to enhance their capabilities. As a result, they are unable to meet the needs of large firms.

**TABLE 11:** Regression analysis of the presence of naive directors.

Variables	Naive (%)			
	(1)	(2)	(3)	(4)
*Size*	−0.026***			
	(−7.96)			
*State*		−0.024***		
		(−3.08)		
*Z_score*			0.025**	
			(2.34)	
*S_score*				0.025***
				(3.08)
*Bnum*	−0.005	−0.043**	−0.006	−0.007
	(−0.26)	(−2.15)	(−0.17)	(−0.20)
*Female*	0.076***	0.079***	0.077***	0.077***
	(4.54)	(4.74)	(3.34)	(3.33)
*Firma*	−0.027**	−0.029**	−0.149*	−0.147*
	(−2.15)	(−2.25)	(−1.92)	(−1.90)
*First*	−0.011	−0.033	−0.011	−0.011
	(−0.49)	(−1.44)	(−1.28)	(−1.39)
*Inde*	0.01	−0.067	0.009	0.01
	(0.14)	(−1.01)	(0.09)	(0.11)
*Growth*	0.016***	0.010*	0.011*	0.011*
	(2.89)	(1.80)	(1.82)	(1.77)
Intercept	1.103***	0.666***	1.100***	1.113***
	(12.67)	(9.19)	(3.78)	(3.83)
Year	Control	Control	Control	Control
Company	Control	Control	Control	Control
N	19809	19809	19677	19677
Adj-R^2^	0.047	0.041	0.211	0.211
*F*-value	15.967***	8.514***	3.17***	3.647***

*, **, *** denote significance level for two-tail test at 10%, 5%, and 1%, respectively.

### Corporate ownership type and naive directors

In terms of corporate ownership type, it can be classified listed companies into state-owned enterprises (SOEs) and non-state-owned enterprises (non-SOEs). If a firm is an SOE, then the variable State equals 1, and 0 otherwise. Compared with Non-SOEs, SOEs have more “political background” and their personnel appointment and assessment differ greatly from that of non-SOEs. In China, the appointment of executives in SOEs is more related to politics, and the recruitment of independent directors is decided by the SASAC (State-owned Assets Monitoring and Administration Commission) rather than the board of directors. Based on this, this paper argues that SOEs are more cautious in selecting independent directors than non-SOEs, and hence they are less likely to select naive directors who lack managerial experience. The regression coefficient between corporate ownership type and naive directors is −0.024 and the *t*-value is −3.08, which is statistically significant at the 1% level, implying that state-owned firms are less likely to select naive directors compared with non-state-owned firms.

### Equity balance degree and the presence of naive directors

Equity balance has a positive impact on the independence of the board. In listed companies, shareholders, who control the appointment of independent directors, have the right to nominate independent directors. If the majority shareholders are dominant, they can control the entire board of directors and try to appoint independent directors with “own party” representatives. In this context, the independence of the board is seriously undermined. Prior research finds that a higher degree of equity balance can enhance the independence of the board and prevent controlling shareholders from manipulating the board of directors (Bennedsen and Wolfenzon, 2000). When a firm’s equity balance degree is higher, other major shareholders have the power to nominate naive directors, thereby enhancing the independence of the board. This paper adopts two indicators to measure equity balance degree: the ownership of the second to fifth largest shareholder scaled by the ownership of the largest shareholder (denoted as Z_score), and the ownership of the second to tenth largest shareholder scaled by the ownership of the largest shareholder (denoted as S_score), finding that equity balance degree is in a positive association with the proportion of naive directors.

Table 11 reveals that the regression coefficients of the indicators of equity balance degree between naive directors are 0.025 and 0.025 respectively, with *t*-values of 2.34 and 3.08, which are statically significant at 5% and 1% levels respectively, indicating that equity balance degree is positively associated with the appointment of naive directors. Therefore, it suggests that major shareholders other than the largest shareholder are more likely to introduce naive directors. The reason may be that in order to have more say on the board of directors, other major shareholders other than the largest shareholder tend to nominate independent directors who can represent their own interests.

## Conclusion and limitations

In China, naive independent directors account for an increasing proportion of the board of directors. However, researchers pay little attention to the influence of naive independent directors on the firm outcome. This paper selects Chinese listed companies during the years 2008−2018 as the research sample and examines the relationship between naive directors and firm performance. It is found that the performance of listed companies that appoint naive independent directors improves significantly. This empirical result suggests that naive independent directors bring benefits to firms. This study further explores the potential channel for this value-enhancing effect and finds that the presence of naive directors can reduce the risk of tunneling of controlling shareholders and financial distress, thus improving corporate performance. Moreover, the paper finds that the association between naive independent directors and firm performance is more pronounced in firms with a lower shareholding of the largest shareholder and firms with lower financial leverage. In further analysis, this paper explores the factors that affect the appointment of naive independent directors and which type of firms benefit more from naive directors and shows that firm size, corporate ownership type, and equity balance degree are important factors affecting the appointment of naive independent directors.

This paper argues that naive directors can enhance the independence of the board, strengthen the monitoring of corporate management and thus improve firm performance. Hence, an appropriate increase in naive directors has a positive effect on the board composition, voting mechanism, and board governance of listed companies. This paper enriches the research on the economic consequences of naive independent directors by investigating their influence on corporate performance and exploring the potential channels.

However, this paper also has its limitations. One of the study’s limitations is that it is based on the data coming from listed companies from China only. It could be interesting to compare the situation in European or American conditions as well. Future research can further examine whether the monitoring role of naive directors still exists in European or American listed companies. In addition, further research can reveal the “black box” of corporate operation of the board of directors and the working mechanisms including nomination, replacement, and pricing of the market of the board of directors by using unique data.

## Data availability statement

The raw data supporting the conclusions of this article will be made available by the authors, without undue reservation.

## Author contributions

GC contributed to the conceptualization, methodology, formal analysis, resources, data curation, and writing original draft preparation. XC wrote, reviewed and edited the manuscript, performed the methodology, and validated the data. PW wrote, reviewed and edited the manuscript, supervised the data, and carried out the project administration. All authors contributed to the article and approved the submitted version.
